# The influence of nutrition on the insulin-like growth factor system and the concentrations of growth hormone, glucose, insulin, gonadotropins and progesterone in ovarian follicular fluid and plasma from adult female horses (*Equus caballus*)

**DOI:** 10.1186/1477-7827-12-72

**Published:** 2014-07-31

**Authors:** Juan Salazar-Ortiz, Philippe Monget, Daniel Guillaume

**Affiliations:** 1 INRA, UMR85 Physiologie de la Reproduction et des Comportements, F-37380 Nouzilly, France; 2 CNRS, UMR6175 Physiologie de la Reproduction et des Comportements, F-37380 Nouzilly, France; 3 Université François Rabelais de Tours, F-37041 Tours, France; 4 IFCE, F-37380 Nouzilly, France

## Abstract

**Background:**

Feed intake affects the GH-IGF system and may be a key factor in determining the ovarian follicular growth rate. In fat mares, the plasma IGF-1 concentration is high with low GH and a quick follicular growth rate, in contrast to values observed in thin mares. Nothing is known regarding the long-term effects of differential feed intake on the IGF system. The objective of this experiment was to quantify IGFs, IGFBPs, GH, glucose, insulin, gonadotropin and progesterone (P4) in blood and in preovulatory follicular fluid (FF) in relation to feeding levels in mares.

**Methods:**

Three years prior to the experiment, Welsh Pony mares were assigned to a restricted diet group (R, n = 10) or a well-fed group (WF, n = 9). All mares were in good health and exhibited differences in body weight and subcutaneous fat thickness. Follicular development was scanned daily and plasma was also collected daily. Preovulatory FF was collected by ultrasound-guided follicular aspiration. Hormone levels were assayed in FF and plasma with a validated RIA.

**Results:**

According to scans, the total number of follicles in group R was 53% lower than group WF. Insulin and IGF-1 concentrations were higher in WF than in R mares. GH and IGF-2 concentrations were lower in plasma from WF mares than from R mares, but the difference was not significant in FF. The IGFBP-2/IGFBP-3 ratio in FF was not affected by feeding but was dramatically increased in R mare plasma. No difference in gonadotropin concentration was found with the exception of FSH, which was higher in the plasma of R mares. On the day of puncture, P4 concentrations were not affected by feeding but were higher in preovulatory FF than in plasma.

**Conclusions:**

The bioavailability of IGF-1 or IGF-2, represented by the IGFBP2/IGFBP3 ratio, is modified by feed intake in plasma but not in FF. These differences partially explain the variability in follicular growth observed between well-fed mares and mares on restricted diets.

## Background

### Follicular growth in mares

In mares, the oestrus cycle has a duration of close to 23 days during the breeding season and exhibits a particular hormonal pattern. The LH concentration increases just before ovulation, peaks 2 days after ovulation and then returns to basal concentrations 5 days after ovulation. The role of this peak is unclear [1]. FSH plasma concentrations are elevated during the follicular phase, decrease one day before ovulation and present a peak after ovulation, which occurs at approximately the same time as the LH peak [2]. Progesterone concentrations increase in the follicular fluid (FF) 2 days before ovulation [3] and in plasma 1 day after ovulation [4]. When the progesterone concentration in the FF is elevated, this indicates impending ovulation. Gerard *et al*. [5] have shown variations in glucose concentrations in FF near ovulation.

In mares, the preovulatory follicle has a higher IGF-1 concentration than the largest subordinated follicle and this increased free IGF-1 concentration is positively correlated with estradiol levels [6]. The increase in the intrafollicular free IGF-1 concentration in the future dominant follicle prepares the follicle to decrease FSH availability and increase LH availability [7]. The other follicles in the follicular wave possess the same capacity for dominance but never reach a similar stage. Thus, the largest follicle alone continues to grow, becomes dominant and ovulates.

### Nutrition and follicular growth

In all mammals, nutrition has a strong influence on cyclicity. In mares, nutrition mainly affects the length of the winter non-ovulatory period. Thin mares demonstrate fewer cycles during the breeding season, while the majority of fat mares exhibit permanent ovarian activity, even during the winter non-ovulatory period. [8].

In saddle mares, Gastal *et al.*[9] found a difference between mares with low body condition and mares with high body condition in terms of the diameter of the preovulatory follicle during the first cycle of the breeding season (45.6 ± 1.4 *vs.* 51.1 ± 1.0 mm, respectively) and during the second cycle (45.1 ± 1.8 *vs.* 51.4 ± 1.0 mm, respectively). In pony mares, follicular growth (measured as the interval from prostaglandin injection to ovulation) was shorter in the unrestricted group than in the diet-restricted group (9.2 ± 0.6 *vs.* 11.6 ± 0.5 days, respectively) [10]. Therefore, in mares, body condition has a strong influence on ovarian follicular growth, and follicular growth is more rapid in fat mares than in thin mares.

### IGFs system and follicular growth

Silva *et al.*[11] have published a review on the respective roles of IGF-1 and 2, IGFBPs and GH in ovarian follicular growth. The insulin-like growth factor (IGF) system plays an important role in ovarian follicular growth and follicular atresia [12]. IGF-1 and IGF-2 bind with high affinity to 6 IGF-binding proteins (IGFBPs) in all biological fluids. IGFBP-1, -2, -4, -5 and -6 are present in all fluids and their molecular weights range from 24 to 35 kDa. In plasma, their levels are negatively regulated (IGFBP-1 and -2) or unaffected by GH. IGFBP-3 is the predominant IGFBP in plasma, where it is found primarily in a 150-kDa form composed of IGF-I or IGF-II and an acid-labile 85-kDa subunit. The concentration of IGFBP-3 is positively regulated by IGF-1 [13]. In the ovaries, the primary action is exerted through IGF-1, which acts on follicular and luteal cells. IGFs increase steroidogenesis via the up-regulation of steroidogenic enzyme expression and activity.

During follicular growth, the IGF system has been implicated in the deviation to ovulation or to atresia. The free IGF-1 concentration in FF increases during follicular growth and selection and is greater in preovulatory than subordinate follicles [14]. This increase in free IGF-1 concentration in FF is due to decreases in insulin-like growth factor binding protein (IGFBP)-2 and -4. The decrease in IGFBP-2 is attributable to decreased synthesis and increased proteolytic activity of pregnancy-associated plasma protein-A (PAPP-A) (in ewes, Monget *et al.*[15] and in mares, Gérard *et al.*[16]). IGFBP-4 is specifically lysed by PAPP-A [17]. FF from dominant follicles in cattle has higher concentrations of steroids and free IGF-1 than large subordinate follicles, but IGF -2 concentrations do not differ between these two follicle types. The levels of IGFBP-2, -4 and -5 in FF are greater in subordinate than in dominant follicles. Yet, expression of IGFBP-3 and -4 mRNA do not differ between dominant or subordinate follicles [18], although PAPP-A mRNA increases in dominant *vs.* subordinate follicles.

In mares, as in other species, the plasma concentrations of IGF-1 and GH are dramatically influenced by individual body condition. In fat mares, the plasma IGF-1 concentration is high and GH concentration is low [8]. Low secretion of GH is mainly attributable to the central negative feedback for IGF-1 exerted by the action of somatostatin. In thin mares, the plasma IGF-1 concentration is low and the GH concentration is high [8]. Reduced secretion of IGF-1 is due to the low availability of GH receptors in the liver, which seems to be dependent on low insulin plasma levels [19] as well as low leptin levels and/or a high adiponectin plasma concentration [20]. In the ovaries, IGF-1 bioavailability plays a critical role in terminating *in vivo* maturation [21], but this bioavailability is most likely modified by feed intake and/or body condition of the mares.

### Study objective

The study objective was to identify the effects of two different long-term food intake treatments on the concentrations of different components of the IGF system in the pre-ovulatory follicles of mares. This study focused on intrafollicular and systemic concentrations of these different components of the IGF system as well as hormones associated with follicular growth. In addition, this study examines whether changes in IGF levels and the IGFBP2/IGFBP3 ratio in the FF of preovulatory follicles in thin mares compared to fat mares could be associated with changes in the bloodstream; that is, whether the IGF system has a certain degree of independence in the FF relative to systemic circulation.

An additional objective was to quantify hormone concentrations in FF, which influence the end of oocyte maturation. These concentrations can be used in oocyte culture medium for *in vitro* maturation and fertilisation to mimic *in vivo* maturation. In different *in vitro* experiments, Marchal *et al.*[22] and Pereira *et al.*[23] used very high levels of different hormones (for example, 400 ng/ml eGH, 200 ng/ml IGF-1, 5 IU/ml pFSH, 10 IU/ml eLH, [23]).

## Methods

### Animals and body condition score

This experiment was conducted with the (INRA) experimental herd in accordance with national animal ethics requirements (French Ministry of Agriculture, Fishing and the Countryside [A37801] and animal experimentation permit 3706). The experiment was performed in August and September and used 2 groups of mares after they completed a previous experiment [8]. The aim of the previous experiment was to quantify the effects of nutritional treatments in generating different body conditions and plasma endocrine levels in the context of the occurrence and length of the winter anovulatory period in mares. At the end of this previous experiment, mare body condition was stabilised for 3 years. Three years prior in the original experiment, cyclic Welsh pony mares were randomly assigned to 3 groups of 10 mares, but only 2 groups were used in the present experiment; one was well-fed (WF) (n = 9 prior to this experiment; one mare from this group was removed from the experiment due to an accidental bone fracture) and the other had a restricted diet (R) (n = 10). The two groups had free access to a paddock during the day and were kept indoors at night. Wheat straw, water and mineral salt were provided *ad libitum*. The WF mares received commercial pellets (1.6 kg/day) supplying 100% of nutritional requirements, while the R mares received an average of 0.74 kg/day of dehydrated alfalfa pellets. Dehydrated alfalfa was chosen to provide a satisfactory protein/energy ratio. This diet provided approximately 60% of the energy requirements calculated for the body weights measured at the beginning of the experiment. Special care was taken with the mares to maintain their health throughout the experiment. Food was distributed twice per day and individually adjusted.

Body weight (BW), body condition score (BCS) and fat thickness were recorded every two weeks. For BCS estimation, the scoring scale used has been previously validated [24], with 0 = emaciated and 5 = obese. Fat thickness was measured in the middle of the hindquarters using ultrasonography (during the same scan as for ovary examination). At the beginning of this experiment, the WF group was 9.5 ± 1.0 years old (mean ± SE) with 323 ± 17 kg for BW, 4.2 ± 0.2 for BCS and 37 ± 1 mm of fat thickness, whereas the R group was 9.8 ± 1.1 years old, 228 ± 25 kg in BW, 1.3 ± 0.2 for BCS and 11 ± 1 mm for fat thickness.

### Ovary ultrasound examinations

Ten days after the previous ovulation (established by P4 assays), ovaries were monitored daily by transrectal palpations and ultrasound examinations. An ultrasound scanner with a 5-MHz linear-array transducer (Aloka SSD-500, UST-5813-5, Tokyo, Japan) was used to measure all follicles with a diameter > 10 mm. For the first set of statistical analyses, the follicles were split into 5 different classes of diameter (>10 mm and ≤15 mm; >15 mm and ≤20 mm; >20 mm and ≤25 mm; >25 mm and ≤30 mm; >30 mm). For a second set of statistical analyses, the volume of the follicle as referenced to a sphere was calculated, and the volumes of each follicle on both ovaries, measured each day, per mare were added.

#### Collection of follicular fluid

One day before expected natural ovulation, follicular fluid (FF) was collected after transrectal palpations and ultrasound examinations, when the dominant follicle was 35 mm in diameter, had apparently stopped growing and appeared to be less hard during palpation than the other follicles. Approximately 3 ml of FF was aspirated as previously described [16,25]. Briefly, a transvaginal ultrasound-guided follicular puncture was used an ultrasound scanner equipped with a 7.5 MHz sector probe (Aloka SSD 900, UST-5820-5, Tokyo, Japan) coupled to a sterile single lumen needle (60 cm in length and 0.6-1.1 mm in outer diameter, Thiébaud Frères, Jouvernex Margencel, France). Approximately 5 to 10 minutes before starting the procedure, mares were given a single intravenous injection of propantheline bromide (2.6 mg/100 kg BW, Sigma, St Quentin Fallavier, France) to ensure rectum relaxation and detomidine chlorhydrate (Domosedan®, 0.57 mg/100 kg BW, Orion Pharma, Espoo, Finland) for sedation. Sterile lubricant was placed on the transducer and the probe was inserted into the vagina, permitting a minimum amount of air into the vaginal vault. The probe was held in the vagina fornix with the transducer facing dorsally. The ovary and the targeted follicle were positioned and stabilised against the vagina wall over the face of the transducer. A second operator then inserted the needle into the needle guide of the transvaginal probe. The ovary was held firmly against the transducer and the needle was moved forward until the image of its tip became visible on the screen, indicating that the vaginal wall and the peritoneum were penetrated. Then, it was advanced until the image of the needle tip was centred within the antrum of the selected follicle, as followed on the screen. FF was collected from the follicle with a 5-ml syringe. The probe and the needle were withdrawn immediately after sampling. FF samples were then immediately aliquoted and frozen on dry ice and stored at 80°C until analysis. After the follicular puncture, the mares were treated with antibiotics (Intramicine, 6.3 ml/100 kg, i.e., benzylpenicillin procaine at 1.67 g/100 kg BW and dihydrostreptomycin at 1,300,000 IU/100 kg BW, i.m.; Ceva Santé Animale, Libourne, France).

### Blood sampling and hormone assays

Blood samples were collected daily at 1:00 PM (5.5 h after animals were fed), beginning 10 days after the previous ovulation and ending 6 days after the puncture session. A supplementary blood sample was collected just after the follicular puncture, between 3:00 PM and 4:00 PM (7.5 h - 8.5 h after animals were fed).

Samples were collected from the jugular vein into heparinised tubes and centrifuged. The plasma was frozen at -20°C and at -80°C for the samples collected just after the puncture session until assays were performed.

FSH, LH, P4 and GH concentrations were assayed in all FF and plasma samples, whereas insulin, IGF-1, IGF-2 and IGFBPs were only assayed from intrafollicular and plasma samples on Day 0 and on Day -6 (Day 0 = day of the puncture session); if Day -6 was not available, the first plasma sample closest to Day -6 was tested (and the denomination Day -6 would still be employed in that case).

### LH and FSH radioimmunoassays

Specific eLH and eFSH radioimmunoassays were previously validated in our lab [3]. For eLH, a polyclonal antibody against equine chorionic gonadotropin (eCG) obtained from rabbits was used and for eFSH, a polyclonal antibody against purified eFSH was also obtained from rabbits. Samples were tested in duplicate, and the location of each sample was randomly assigned. To calculate concentrations, standards ranging from 0.05 ng/ml to 800 ng/ml were prepared for LH in the plasma of a mare that was in a period of winter inactivity and treated with a progestagen (Regumate®, Hoechst Roussel Vet, Romainville, France) and for FSH, standards were prepared from\ plasma obtained from a hypophysectomised mare. Free plasma and 3 other blood samples collected from other mares were routinely tested as quality controls for every box of 100 samples. After 24 h of incubation with the first antibody at 4°C, immunoprecipitation was performed with a solution of antiserum anti-rabbit gamma immunoglobulin obtained from sheep and polyethylene glycol. After centrifugation, the supernatant containing free-labelled and unlabelled hormone was removed from the test tube, and the radioactivity was determined using a gamma counter. For LH, the intra-assay coefficients of variation for samples containing 0.76 or 6.00 ng/ml of eLH were 4 and 10%, respectively. For FSH, the intra-assay coefficients of variation for samples containing 4.35 or 9.00 ng/ml of eFSH were 9 and 11%, respectively.

### GH, insulin and glucose assays

A specific equine GH radioimmunoassay, insulin radioimmunoassay and a glucose chemical assay were previously validated in our lab [8]. GH and insulin are classical RIAs that use a second antibody to precipitate the antibody-antigen complex absorbed on polyethylene glycol as LH or FSH. For GH, the first antibody was obtained in rabbits immunised against recombinant equine GH. For insulin, the first antibody was obtained in guinea pigs (Sigma-Aldrich Corporation, Saint Quentin Fallavier, France). For GH, the intra-assay coefficients of variation for references containing 26, 57 or 94 ng/ml of eGH were 10, 7 and 9%, respectively. For insulin, the intra-assay coefficient of variation for a reference containing 2.7 ng/ml of eInsulin was 6%. For glucose, a specific glucose oxidase method was used with a Coulter glucose analyser (Beckman®, Palo Alto, CA, USA). The rate of oxygen consumption was directly proportional to the glucose concentration. For a standard of 450 mg/dl, the stated error was less than 3%.

### P4 assay

A specific P4 radioimmunoassay was previously validated in our lab, using a polyclonal antibody against P4 obtained from rabbit and tritiated P4. For each of the 6 references included in the assay, the coefficient of variation was less than 10%.

### IGF-1 and IGF-2

IGF-1 and IGF-2 were assayed with a previously validated RIA [26]. Briefly, the IGFs were separated from their binding proteins by acidification (25 μL of samples in 2 ml of chlorhydric acid, 0.01 N). After, samples were ultrafiltered through an Amicon® Centricon® centrifugal filter with a molecular weight cut-off of 30 kDa (Millipore Corporation, Bedford; MA, USA) with two centrifugation steps. After lyophilisation, samples were dissolved in 500 μl binding buffer (0.03 M NaH_2_PO_4_, pH 7.4, 0.02 M EDTA, 0.2 g/L protamine sulphate, 0.2 g/L sodium azide, 0.05% Tween 20). A classical RIA was performed with these recovered samples using an antibody against human IGF-1 or IGF-2 (Amano Pharmaceutical Co., Nagoya, Japan) with human IGF-1 or 2 as a standard. Thereafter, free and bound IGFs were separated with a buffer containing active charcoal and centrifugation.

### IGFBP assays

The IGFBPs were assayed using western ligand blotting as previously described [16]. SDS-polyacrylamide gel electrophoresis (SDS-PAGE) was performed under non-reducing conditions [27]. Samples (2.5 μL of FF or plasma) were supplemented with 8 μl of Laemmli buffer (for 10 ml: 2.5 ml of 0.5 M Tris–HCl, pH 6.8, 2.0 ml glycerol, 4.0 ml of 10% (w/v) SDS and 0.5 ml of 0.1% bromophenol blue) and then heated at 95°C for 3 min. Samples were subjected to 12% SDS-PAGE at 80 V for 20 min until the dye had left the gel. After electrophoresis, each gel was soaked in transfer buffer (50 mM Tris, 0.4 M glycine, 20% methanol and 0.1% SDS) and placed on a single Immobilon-PVDF membrane (0.45 μm; Millipore Corporation, Bedford, MA, USA) equilibrated in transfer buffer. Each gel-PVDF membrane assembly was sandwiched between two sheets of Whatman filter paper saturated with transfer buffer and two sponge pads. These "sandwiches" were electroblotted under constant voltage at 40 V overnight at 4°C. After transfer, membranes were treated first for 10 min with PBS (0.01 M, pH 7.4) containing 0.1% Nonidet P-40 (non-ionic detergent), second for 3 h with 5 g/L gelatin and last for 10 min with 0.1% Tween 20 (non ionic detergent) to saturate the membranes. Membranes were incubated overnight at 4°C with ^125^I-IGF-2 in binding buffer. Thereafter, the membranes were washed four times at 4°C in PBS (0.01 M, pH 7.4) containing 0.1% Tween 20. The blots were air dried and exposed to -80°C for 48 h for autoradiography (Hyperfilm MP; Amersham Biosciences, Buckinghamshire, UK). Membranes were exposed to a storage phosphor screen for 24 h. The screen was then digitalised with a Molecular Dynamics Storm 640 Phosphor Imager.

### Statistical analysis

All statistical analyses were carried out using SAS software (SAS Institute Inc., Cary, NC, 27513, USA).

The number of follicles in each diameter class was compared each day between the R and WF groups using an exact unilateral Mann–Whitney Wilcoxon test purchased with the "NPAR1WAY" procedure.

Body weight, fat thickness and BCS were compared using analyses of variance (ANOVA). The FSH, LH, P4, GH and follicle volume curves were analysed by ANOVA using a repeated measures model in the ‘GLM’ procedure [28] (Little *et al.* 1998) after log transformation. On the day of the puncture, FSH, LH, P4, GH, insulin, IGF, IGFBP2 and IGFBP3 levels and the ratio of IFBP2/IGFBP3 were compared between plasma and FF using ANOVA after log or arcsin (square root) transformation for the ratio of IFBP2/IGFBP3. The effects were considered significant when p ≤ 0.05.

## Results

### Puncture session and follicular growth

All mares (n = 19) were punctured one day after their dominant follicle achieved a diameter equal or larger than 32 mm. The diameters of the punctured ovulatory follicles did not differ between the WF and R groups (35 ± 1 mm versus 34 ± 1 mm, respectively).

**Figure 1:**
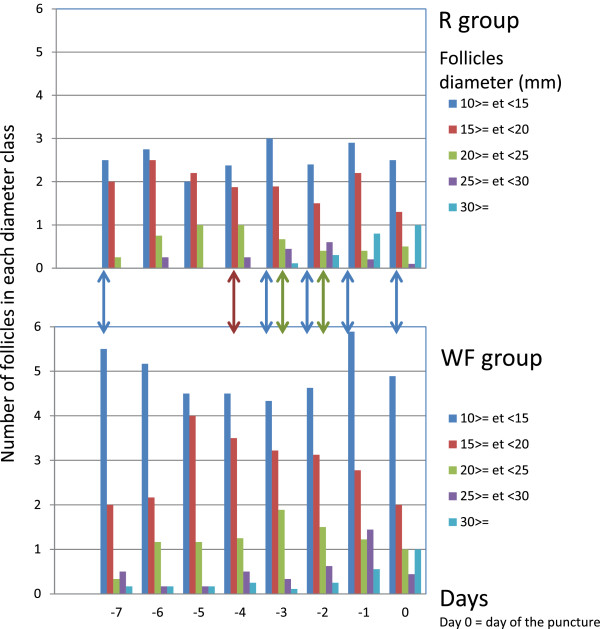
**Mean numbers of follicles in each class diameter on each day for the 2 experimental groups.** The vertical arrows (with the same colours as the follicle categories) indicate when the number of follicles is significantly higher according to an exact unilateral Wilcoxon test with p < 0.05.

**Figure 2:**
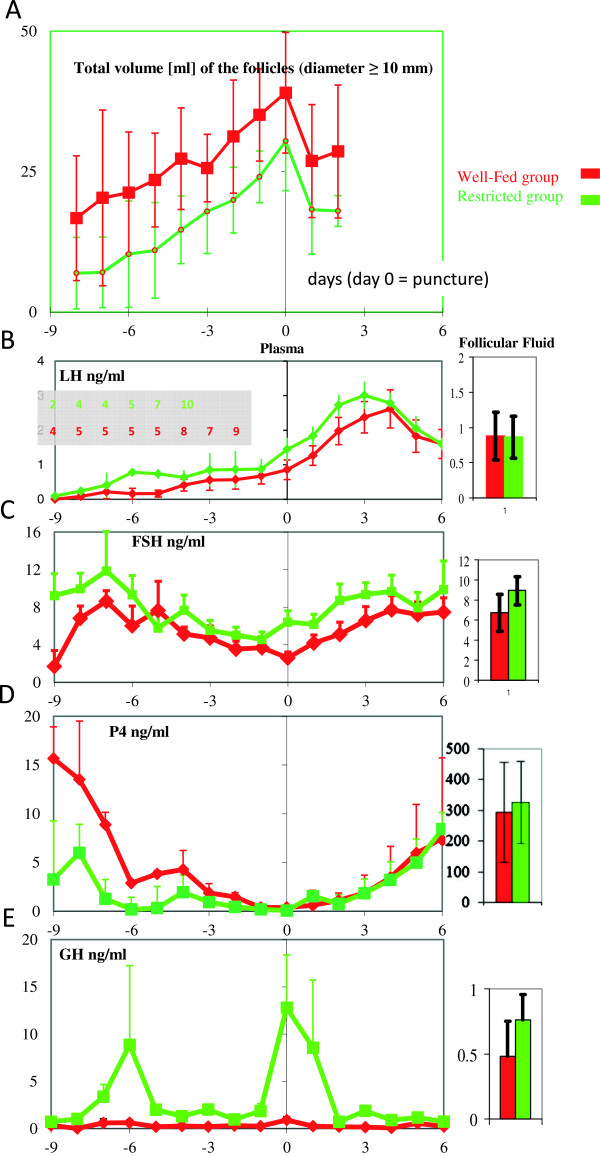
**Follicular growth and the daily variation of FSH, LH, GH and P4 concentrations in plasma and FF in WF and R mares as a function of the day of puncture (Day 0). A)** Sum of the volumes of all the measured follicles (diameter > 10 mm), including the dominant follicle, extrapolated as a sphere. **B)** LH: On the day of the puncture, LH concentrations were not significantly different between treatment groups or between FF and plasma. The table included in the figure gives the number of mare plasma samples assayed before all mares are collected. **C)** The FSH concentration was significantly higher in plasma from R mares than in plasma from WF mares, but this difference was not significant in FF or between plasma and FF. **D)** P4: On the day of puncture, P4 concentrations were not different between treatment groups but were significantly higher (p > 0.0001) in FF than in plasma. **E)** GH: On the day of puncture, GH concentrations were significantly higher (p = 0.008) in mares with restricted diets, but no significant difference between plasma and FF was observed.

The number of follicles appeared to be significantly lower in the R group than in the WF group for the low diameter categories (10–14 and 15–19 mm). The details of each category on each day are presented in Figure 1. In the 2 groups, the sum of the volumes of all measured follicles (diameter > 10 mm), including the dominant follicle, estimated similar to a sphere, is presented in Figure 2A Despite these different follicular populations between R and WF groups, the growth of the dominant follicle (i.e., > 25 mm size categories) was similar in both groups. Its growth rate and maximum diameter before puncture did not vary between the WF and R groups (2.3 mm/day and 35 ± 1 mm *vs.* 2.3 mm/day and 34 ± 1 mm, respectively). All the WF and R mares ovulated 3 ± 1 days and 2 ± 1 days, respectively, after follicular puncture.The mean concentrations of LH, FSH, P4, and GH in plasma and in FF from WF and R groups are shown in Figures 2B-E. Unfortunately, the blood sampling collection began too late in a few mares. The number of blood samples assayed is presented in a table included in Figure 2B. In both the WF and R groups, circulating plasma LH concentrations rose gradually before the puncture and reached a peak at 3 or 4 days after puncture. One WF mare was removed from this analysis due to its late LH surge. The LH surge of the WF group tended to be lower and delayed when compared with the R group. The day of the puncture, LH concentrations did not differ significantly between FF and plasma or between WF and R groups.

In both WF and R treatment groups, the mean circulating plasma FSH concentration rose gradually from 6 days before ovulation to a plateau, decreased before puncture and reached a peak at 3 or 4 days after puncture. Circulating FSH concentrations were statistically similar between R and WF treatment groups. On the day of the puncture, FSH concentrations did not differ significantly between FF and plasma and between WF and R groups.

In some WF mares, P4 secretion due to the previous *corpus luteum* was not completely finished at the beginning of blood collection. However, P4 FF concentrations were higher than in plasma (P < 0.0001), but no differences in P4 concentrations after puncture were observed for plasma or FF between WF and R groups.

**Figure 3:**
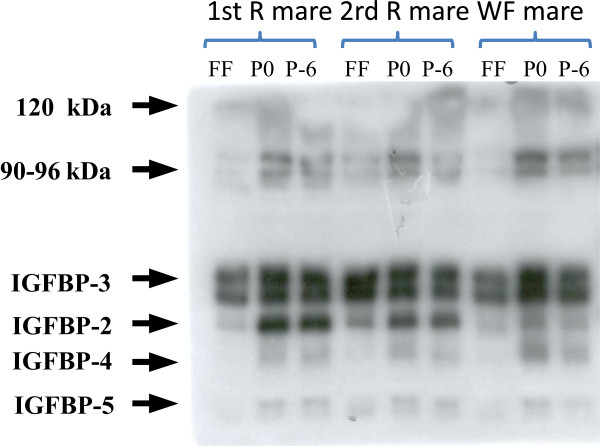
Representative western ligand blot of IGFBPs detected in FF or in plasma 6 days prior to the puncture session (P-6) and the day of the puncture (P0) in 2 group R mares and in 1 group WF mare.

**Figure 4:**
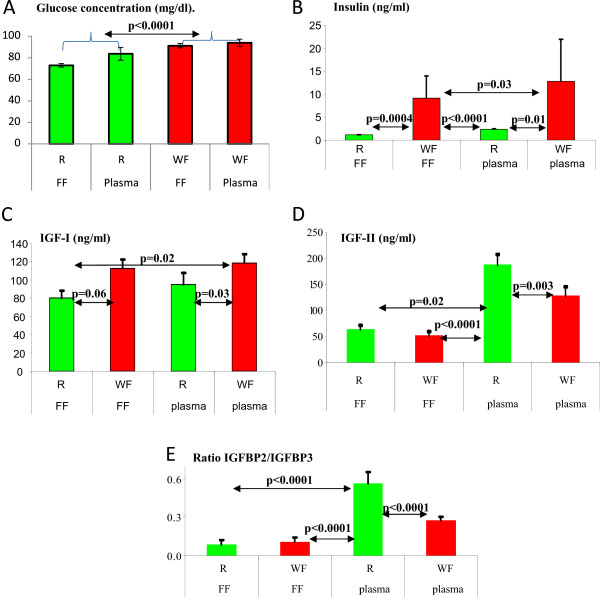
**Plasma and FF levels of glucose, insulin, IGF-1 and IFG-2 and IGFBP2/IGFBP3 ratios in the WF and R treatment groups. A)** Glucose concentrations, measured via the glucose oxidase method on the day of puncture are globally higher (p < 0.0005) in well-fed mares than in those receiving a restricted diet, although no global difference appeared between follicular fluid and plasma. **B)** Insulin: On the day of puncture, insulin concentrations were higher (p < 0.0001) in the well-fed treatment group compared to the restricted-diet group, but no significant difference was present between FF and plasma. **C)** IGF-1: On the day of puncture, IGF-1 concentrations were globally higher (p < 0.0061) in the well-fed treatment group compared to the restricted diet group, but there was no significant difference between FF and plasma. **D)** IGF-2: On the day of puncture, IGF-2 concentrations were higher (p < 0.0028) in the restricted diet treatment group compared to the well-fed group and in plasma compared to FF. **E)** IGFBP2-to-IGFBP3 ratio: On the day of puncture, the IGFBP2-to-IGFBP3 ratio, treatment group effects, FF/plasma effects and the interaction between treatment groups and FF/plasma were significant (p < 0.03).

Overall, eGH concentrations were significantly higher (P < 0.05) in the R group than in the WF group. However, in each group no difference was observed between plasma and FF for the day of the puncture.An example of a western ligand blot is shown in Figure 3. Concentrations of IGFBP-4 and 5 appear to be very low in the FF, near the limits of quantification of our method. These two IGFBPs were excluded from the statistical analyses. The mean concentrations of glucose, insulin, IGF-1, IGF-2 and the ratio of IGFBP-2 to IGFBP-3 in plasma and FF of the WF and R groups are shown in Figures 4A-E. The area of the IGFBP2 on the Phosphor Imager appeared also significantly higher in R mares than in WF mares (p = 0.027) and higher in plasma the day of the puncture than in FF (p < 0.0001). For IGFBP3, the group effect was not significant, but the area was higher in plasma the day of the puncture than in FF (p = 0.0204).

Glucose concentrations (Figure 4A) were globally higher in the WF group than in the R group (P < 0.0005). In the WF group, this concentration was similar in plasma and FF (94.5 ± 3.4 *vs.* 93.1 ± 4.6 mg/dl, respectively). In contrast, in the R group, glucose concentrations in plasma were significantly (p < 0.05) higher than in FF (84.1 ± 5.9 *vs.* 72.6 ± 1.6 mg/dl, respectively).One mare was removed from the WF group due to its abnormally elevated insulin concentrations in plasma Day 0, in plasma Day -6 and in FF (25 ng/ml, 60 ng/ml and 48 ng/ml, respectively). In the R group, insulin concentrations did not significantly differ between FF and plasma Day 0 or plasma Day -6 (data from Day -6 not shown). These insulin concentrations were lower in the R group than in the WF group (Figure 4B).For IGF-1 (Figure 4C), the R group had lower concentrations of IGF-1 in plasma and in FF than in WF mares (p < 0.05). In both groups, concentrations of IGF-1 tended to be higher in plasma than in FF (but this difference was only significant between plasma from the WF group and FF from the R group).

Contrary to IGF-1, IGF-2 concentrations were higher in plasma and in FF in R mares than in WF mares. IGF-2 concentrations from WF mares also significantly differed between plasma Day 0, plasma Day -6 and FF. Concentrations of IGF-2 from FF were lower than concentrations from plasma in both groups, similar to IGF-1, whereas circulating concentrations of IGF-2 were higher on Day 0 than Day 6 in the R group.

Western ligand blots with [^125^I]-IGF-2 revealed six forms of IGFBPs present in plasma and FF in the WF and R groups (Figure 3). IGFBP-2 and IGFBP-3 are the major binding proteins present in plasma and FF. Only these two binding proteins were quantified. As depicted in Figure 2E, in plasma the IGFBP-2-to-IGFBP-3 ratio for R mares was higher than for the WF mares but was similar in FF. No variation occurred between Day 0 and Day 6 in WF or R mares.

## Discussion

### Moment of puncture and progesterone concentrations

Eighteen of the 19 punctures for FF were performed when circulating LH had already started increasing. P4 levels in plasma were near zero but were very high in FF in all mares, which agrees with a previous study [2]. For LH and P4, the punctures were performed on the preovulatory follicles just before ovulation. Systemic concentrations of LH continued to increase even after the puncture session and peaked 1 or 2 days after ovulation, as expected. This confirms that follicular maturation was not affected by puncture, only delayed.

In Figure 2D, P4 values were higher in WF mares than in R mares 4 days before the punctures. This is the consequence of the follicular growth phase, which was shorter in WF than in R mares [10]. Thus, the previous progesterone phase was nearly completed in the WF groups at the beginning of this experiment.

### Follicular development

Chronic poor nutrition led to decreases in the classes of follicle populations (10–14 mm, 15–19 mm and 20–24 mm in diameter). However, the diameter of the pre-ovulatory follicle was not affected by chronic poor nutrition. Taken together, these results indicate that in mares, chronic poor nutrition affects the recruitment of follicles and the selection of the dominant follicle but not the dominance phase. Thus, previous results in mares were verified [9,10]. Comparable results were also observed in cattle [29-32]. In polyovulatory species, a short period of improved nutrition (flushing) is sufficient to increase ovulation rate (sheep [33], pigs [34]). Treatment with exogenous GH appears to counteract the effects of restricted feed intake on follicular growth rate [35,36].

### Glucose concentrations

Glucose concentrations were higher in the WF treatment group than in the R group, which is consistent with previous data on these ponies [8] and were unaffected by the nature of the sample. In FF, concentrations were similar to those previously reported for mares [5]. Glucose availability is a limiting factor for ovarian function [37]. The inhibition of glycolysis in cows with the administration of 2-deoxy-D-glucose induced the failure of oestrus, ovulation and the formation of a new *corpora lutea*[38]. On the other hand, a short-term infusion of glucose in ewes stimulated follicular growth [39].

### Insulin concentrations

Insulin acts in all compartments of the ovary via its own receptor. It stimulates steroidogenesis and sensitises follicular cells to the action of FSH and LH (for review: [40]). Our experiment with insulin concentrations showed a large difference between the 2 diet treatments and no difference between FF and plasma. This difference is due to the postprandial peak of insulin, which was previously demonstrated for these ponies [8]. The absence of a difference in insulin concentrations between plasma and FF is consistent with the results on cyclic gilts [41].

### Gonadotropin concentrations

During seasonal ovarian inactivity in ewes [42], as in mares [8], the effects of nutrition on LH secretion have been characterised, but during breeding season, this effect has not been well demonstrated. In heifers, during cyclicity, a situation of severe undernutrition results in anovulation and LH secretion is affected [43], but this is not the case in many other studies (pigs [34], sheep [44], heifers [31]). In mares, McManus and Fitzgerald [45] reported no change in gonadotropin secretion in mares deprived of food for 24 h. Similarly, we did not find any alteration in the secretion of LH in the R group compared with the WF group.

The observed difference between the 2 treatment groups for FSH confirms previous results for equines [9,10]. This low difference appears to be due primarily to the difference in follicular growth. The intense follicular growth in WF mares induced more oestrogen secretion, which induced negative feedback for FSH secretion.

### GH levels

In our experiment, plasma GH levels were higher in the R group than in the WF group. Previous results with these pony mares were confirmed [8], and this is a general rule for mammals, with the exception of rodents [46]. Responsiveness to GH is dependent upon the expression of GH receptor (GHr) [47] expressed in hepatocytes [48]. A poor diet induces GH resistance characterised by high GH and low IGF-1 plasma levels [49], reduced hepatic synthesis of IGF-1 and the secretion of insulin [50]. Low IGF-1 levels induced low negative feedback actions on GH secretion [46]. Circulating concentrations of IGF-1 and insulin consistently decline in sheep and cattle during fasting [51,52]. In ewes, a glucose perfusion boosts insulin and IGF-1 secretion [39].

### IGF-1 and -2 levels

IGF-1 concentrations in R mares were lower than those in WF mares, agreeing with the results of several previous studies (in cattle: [31,12]; in mares: [53,54], [8]). The low increase in systemic IGF-1 levels at the preovulatory stage is consistent with previous studies (mares: [55,54]; heifers: [12,31]).

In our experiment, a difference between the two groups for IGF-1 concentrations was found but not between plasma and FF. These results suggest that the local secretion of IGF-1 in the ovary is negligible and the greater quantity of IGF-1 found in FF is produced by the liver and routed by the blood to the ovaries. The correlation between plasma and FF concentrations for IGF-1 was shown for large follicles in sheep [26,56] and in mares [54]. The bioavailability of the IGFs in the follicle is influenced by the relative proportions of IGFBPs [26,57,58], which have different affinities for their specific ligands [59]. In serum, 90% of IGF-1 circulates in the large 150 kDa-complex form composed of IGF-1, IGFBP-3 and an acid labile subunit [60]. The remaining IGF-1 binds to other IGFBPs. The large cut-off at the basal membrane of follicles (500 kDa) allows all complexes made of IGFs and IGFBPs to freely circulate between plasma and FF. However, the follicular basal membrane could be a barrier to the crossing of growth factors from the theca interna to the granulosa and *vice versa*. The treatment of cattle with GH leads to an increase in IGF-1 levels in both compartments [61], but a short food restriction in heifers only affects plasma and not FF IGF-1 concentrations [51]. These observations suggest that the greater quantity of intrafollicular IGF-1 is coming from the blood. As we have previously shown in these ponies [8], the regulation of IGF-1 secretion after the change in feeding appears to exhibit an important delay.

Plasma IGF-2 concentrations in our study were in the same range as those reported by Bridges *et al*. [55] and did not vary significantly. Plasma and FF IGF-2 concentrations in mares were lower than IGF-1 concentrations, which seems to differ from other species [6].

Plasma IGF-2 concentrations were higher in R mares than in WF mares, whereas FF concentrations were only slightly different. In sheep, Oldham *et al*. [62] have shown that variations in plasma IGF-2 concentrations as a function of feed intake are age-dependant: fasting increased IGF-2 concentrations in adult sheep but not in young sheep.

### The IGFBPs

Nutrition modifies plasma and FF concentrations [63] of IGFBPs, which control the bioavailability of the IGFs. IGFBP-3 binds IGF-1 or -2 in a ternary complex containing the acid-labile subunit [64,65].

IGFBP-4 and -5 are found in small amounts, particularly in FF close to ovulation, which is in agreement with previous results [66,55]. Therefore, the level of IGFBP-2 is higher in the R group, and the level of IGFP-3 does not differ between the 2 groups. The complex formed between IGFBP-3 and the IGFs is less degraded than the other complexes. This clearly demonstrated that IGF bioavailability is decreased in long-term restricted mares, which is mainly attributable to the increased IGFBP-2. In plasma, the ratio of IGFBP-2 to IGFBP-3 was largely increased by chronic restriction of feed intake but not in FF. The absence of a difference in FF is due to IGFBP-3 levels, which were lower in FF than in plasma. These results indicate that in our study, or in [41], a long-term low-feed intake increases IGFBP-2 levels in plasma [41]. Consequently, chronic poor nutrition does not affect IGFBP concentrations in equine preovulatory follicles, whereas these concentrations increase in plasma. The variations in IGFBPs are better indicators of IGF bioavailability than IGF concentrations only. During follicular growth, the decrease in IGFBP-2 and -4 in preovulatory follicles is mainly attributable to the proteolytic degradation of pregnancy-associated plasma protein-A (PAPP-A) [16,17]. Thus, PAPP-A may be involved in the maintenance of low levels of IGFBPs in equine preovulatory follicles, even in cases of chronic undernutrition.

## Conclusions

In conclusion, in this work we have demonstrated that long-term intake in mares modified the systemic concentrations of insulin IGF-1 and -2. Their bioavailability in the ovaries was altered mainly due to modifications in IGFBP-3 concentrations. These diets also altered the number of recruitable follicles but did not appear to alter the terminal growth of the dominant follicle.

## Abbreviations

BCS: Body condition score; FF: Follicular fluid; FSH: Follicle stimulating hormone; g: Gravitational acceleration; GH: Growth hormone, also known as somatotropin; GLM: General linear model procedure: an analysis of variance in SAS software; IGF-1: Insulin-like growth factor 1; IGF-2: Insulin-like growth factor 2; INRA: French National Agronomical Research Institute; LH: Luteinising hormone; MADC: Horse digestible crude protein (INRA 1997); PAPP-A: Pregnancy-associated plasma protein-A; R: Experimental group of mares with restricted diets; RIA: Radio-immuno-assay; UFC: Horse feed unit (INRA 1997); WF: Experimental group of well-fed mares with feed intake calculated to maintain good body condition.

## Competing interests

The authors declare that they have no competing interests.

## Authors’ contributions

JSO performed the glucose, insulin and GH assays and wrote the manuscript. PM validated the IGF system quantification. DG supervised the work, developed and validated the different FSH, LH, GH, and insulin RIAs, performed the ovary ultrasound examinations and part of the collection of follicular fluid, performed the statistical analysis with SAS and wrote the manuscript. All authors read and approved the final manuscript.

## Authors’ information

During the experiment, JSO was a PhD student. He is now "Profesor Investigador Adjunto at Colegio de Postgraduados", Campus Córdoba, MEXICO. PM and DG are permanent researchers.
